# Editorial: Current status of neural networks that subserve emotion and cognition – unraveling the complex brain through multidisciplinary contributions in awake brain surgery

**DOI:** 10.3389/fpsyg.2025.1627165

**Published:** 2025-05-30

**Authors:** Isabel Martín-Monzón, Lucas Alverne Freitas de Albuquerque, Andrés Cervio, Lucia Crivelli

**Affiliations:** Department of Experimental Psychology, Faculty of Psychology, University of Seville, Seville, Spain

**Keywords:** awake brain surgery, neural networks, connectome, emotion, cognition, neuropsychology and neurosurgery, neuropsychological assessment, intraoperative mapping

The human brain is increasingly conceptualized not as a Research Topic of discrete, modular regions, but as a dynamic and integrated system. Nowhere is this paradigm shift more evident than in the evolving practice of awake brain surgery. As this field advances, so too does our understanding of the neural substrates underpinning cognition and emotion—processes once considered separable, now recognized as deeply entangled within large-scale, interactive networks (Herbet and Duffau, 2020). This Research Topic reflects a growing consensus: that preserving brain function means more than avoiding damage to the previous known as “eloquent areas”—it demands a deeper engagement with the complexity of neural organization, and with the lived experiences of the patients undergoing surgery.

The clinical implications of this shift are far-reaching. For instance, functions previously underestimated in neurosurgical contexts—such as affective prosody, social cognition, and spatial awareness—are gaining overdue recognition. Mapping protocols have traditionally focused on language and motor domains, often to the exclusion of these right-hemisphere-dominant processes. Yet, as Martín-Monzón et al. demonstrate in their systematic review, robust intraoperative tools to evaluate such functions are not only feasible but urgently needed. Their analysis calls for more comprehensive, balanced protocols that incorporate both hemispheres in preoperative planning, reminding us that the emotional and interpersonal capacities housed in the right hemisphere are no less essential to personhood than speech or movement.

Parallel efforts aim to refine how cognition is assessed in the surgical setting. The perioperative environment demands assessments that are both efficient and sensitive—a balance exemplified by the protocol proposed by Ohy et al.. Their brief neuropsychological battery addresses a practical gap, offering clinicians tools to monitor a range of cognitive domains while respecting time and patient endurance. More than a technical solution, this approach reflects a broader ethical imperative: to understand and preserve not just “function,” but the unique cognitive profiles of individual patients.

At the theoretical level, the limitations of localizationist thinking are increasingly evident. Higher-order functions such as metacognition, social understanding, or the sense of self do not localize neatly to any one cortical area. As Martín-Fernández et al. argue, these capacities emerge from distributed, recursive interactions within complex neural systems. Drawing from systems theory, their perspective invites neurosurgeons and neuroscientists alike to move beyond static models, embracing dynamic network frameworks that account for context, plasticity, and interdependence. This view challenges not only how we map the brain, but how we define the boundaries of cognitive neuroscience itself.

Structural connectivity studies continue to elucidate the anatomical scaffolding of these networks. The complexity of the inferior fronto-occipital fasciculus (IFOF) represents one of the frontiers of surgical and functional knowledge, and recent work by Nogueira et al. in a beautiful work of anatomical dissection and functional review brings valuable information and highlights its role in semantic and multimodal integration. Their findings reinforces that associative pathways like the IFOF support high-level cognitive functions through long-range cortical communication, underscoring the need to protect such tracts during surgical resection. Understanding white matter connectivity is thus not only a matter of academic interest but a cornerstone of functionally informed neurosurgery.

What emerges from these multidisciplinary contributions is a shared commitment to reconceptualizing the brain not in terms of isolated abilities, but as a landscape of potential shaped by interaction. Cognitive and emotional processes cannot be easily disentangled from the networks that support them, nor from the people who experience them. In this context, awake brain surgery becomes not just a clinical procedure but a window into the architecture of the mind—a unique opportunity to observe cognition in action and to intervene with unprecedented precision.

However, the human dimension must never be overlooked. Patients are participants in a deeply personal process and the psychological toll of being conscious during surgery—of performing tasks that reflect one's identity and agency—cannot be overstated. This raises not only logistical and ethical challenges, but also opportunities: to develop practices that are truly patient-centered, to co-construct surgical goals with patients, and to redefine success in terms that go beyond extent of resection.

In this light, the integration of advanced neuroimaging, refined mapping techniques, and systems-level theory is not merely technical progress—it is an ethical commitment. Each methodological advancement reflects a deeper respect for the complexity of human cognition, and for the responsibility borne by those who operate at the intersection of brain and behavior.

This Research Topic exemplifies how collaboration across disciplines—neurosurgery, neuropsychology, cognitive science, and theoretical modeling—can yield transformative insights. The boundaries between research and care, between science and ethics, between structure and experience, are not lines to be enforced but bridges to be built. As we move forward, the challenge will be to maintain this integrative momentum: to refine intraoperative techniques, to standardize assessment without sacrificing individuality, and to continue asking not only how the brain works, but what kind of lives we enable when we intervene upon it.

By embracing complexity—biological, cognitive, and human—we can continue to improve outcomes not just in terms of function, but in terms of meaning. And in doing so, we draw closer to understanding the full richness of the brain's networks, and of the people whose lives they sustain.

The multidisciplinary contributions in this Research Topic underscore the necessity of a holistic approach to neurosurgery—one that integrates neuroanatomical, psychological, and ethical considerations. By embracing a network-based understanding of brain function and leveraging advanced imaging and assessment tools, clinicians can enhance surgical precision and patient outcomes. The anatomical clarification of tracts like the IFOF (Nogueira et al.), the advocacy for right-hemisphere mapping (Martín-Monzón et al.), the development of pragmatic neuropsychological tools (Ohy et al.), and the theoretical reconceptualization of cognition as dynamic and non-local (Martín-Fernández et al.) collectively represent key steps in this direction. Future research should continue to explore the dynamic interplay of neural networks, refine intraoperative mapping techniques, and address the psychological aspects of awake surgery. Through such endeavors, we move closer to unraveling the complexities of the human brain and improving the lives of those undergoing neurosurgical interventions.

## References

[B1] Herbet G. Duffau H. (2020). Revisiting the functional anatomy of the brain: toward a meta-networking theory of human cognition. Physiol. Rev. 100, 1181–1209. 10.1152/physrev.00033.2019 32078778

